# Site-specific prolongation of repolarization prevents postmyocardial infarction tachycardia

**DOI:** 10.1016/j.hroo.2023.06.003

**Published:** 2023-06-08

**Authors:** Ryan P. O’Hara, Veronique M.F. Meijborg, Pouya Jelvehgaran, Jeanne van der Waal, Gerard J.J. Boink, Natalia A. Trayanova, Ruben Coronel, Bastiaan J. Boukens

**Affiliations:** ∗ Institute for Computational Medicine and Department of Biomedical Engineering, Johns Hopkins University, Baltimore, Maryland; † Heart Center, Department of Clinical and Experimental Cardiology, Amsterdam UMC, University of Amsterdam, Amsterdam, The Netherlands; ‡ Department of Medical Biology, Amsterdam UMC, University of Amsterdam, Amsterdam, The Netherlands; § Fondation Bordeaux Université, Inserm, U1045 and Université de Bordeaux, Bordeaux, France; ¶ Department of Physiology, Cardiovascular Research Institute Maastricht, Maastricht University Medical Center, Maastricht, The Netherlands

**Keywords:** Antiarrhythmic therapy, Digital twin, Myocardial infarction, Reentry, Ventricular tachycardia

**Key Findings**

▪ Prolongation of repolarization in myocardium adjacent to infarcted myocardium may prevent reentry and postinfarction ventricular tachycardia.
▪ Local injection of sotalol does not cause conduction delay or myocardial injury.
▪ We speculate that site-specific targeted antiarrhythmic therapy will provide a nondestructive, mechanism-based treatment of arrhythmic substrates, to a degree not attained with drugs or ablation and without their side effects.

Antiarrhythmic drugs that aim to prolong refractoriness by reducing repolarizing currents are often used to prevent postinfarction ventricular tachycardia (VT). However, these drugs prolong action potential duration throughout the heart and may trigger polymorphic VT by prolonging repolarization in areas where it already is long. Here we investigate whether site-specific prolongation of repolarization in myocardium adjacent to the infarcted myocardium mitigates reentry and prevents postinfarction VT.

**Figure 1:**
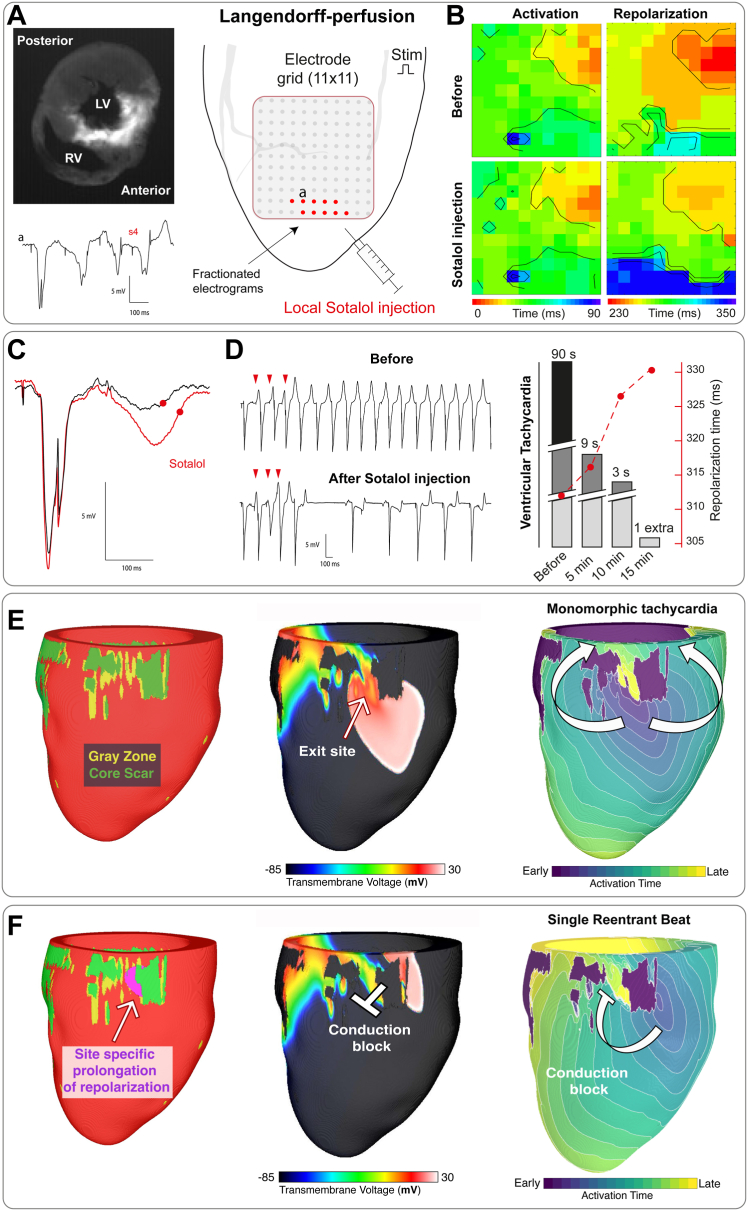
Site-specific prolongation of repolarization prevents postinfarction tachycardia. **A, left:** Transversal section of the pig heart at the level of the apex showing infarcted myocardium based on autofluorescence. **Middle:** Schematic drawing of the left anterior wall indicates the location of the multielectrode and the stimulus electrode. **Right:** Tracing shows pronounced fractionated potentials initiated by the third premature stimulus (s4), which were only present at the location of the *red dots* in the grid. **B:** Map showing the activation and repolarization sequence before **(top)** and after **(bottom)** sotalol injection at basic cycle length (s1). Note local prolongation of repolarization (±25 ms) at the location where fractionated potentials were present. **C:** Local electrograms before and after local injection of sotalol. **D:***Upper trace* shows a monomorphic tachycardia induced by premature stimulation (s1-s2-s3-s4). After local sotalol injection, only a single extra beat was induced *(lower trace). Bar graph* shows the decrease in arrhythmia inducibility. *Superimposed red line* indicates the simultaneous increase in local repolarization time of S1 in the area with fractionated potentials. **E, left:***In silico* reconstruction of the left ventricular septal and free wall showing noninfarcted myocardium in *red,* gray zone in *green,* and scar in *yellow.***Middle:** Potential distribution shows the activation front exiting the infarcted myocardium resulting in sustained reentry, of which the first cycle is shown at the **right. F, left:** Region in *purple* shows prolonged repolarization (±50 ms) near the exit site. **Middle:** Activation front exiting the infarcted myocardium at a different location than before site-specific prolongation of repolarization, resulting in only a single reentrant beat **(right).** LV = left ventricle; RV = right ventricle.

We generated myocardial infarction in a 2-year-old male pig by 90-minute occlusion of the left anterior descending coronary artery. Six weeks later, we performed epicardial mapping during Langendorff perfusion as reported previously.^1^ Programmed stimulation induced reentry and monomorphic VT. We locally injected 0.5 mL sotalol (10 μM) at the site with fractionated electrograms to prolong repolarization. Direct injection of sotalol into the myocardium did not cause injury as evidenced by the absence of ST elevation in the local electrograms which was further supported by similar epicardial activation patterns before and after sotalol injection. Fifteen minutes after sotalol injection, VT could no longer be induced (Figure 1A-D).

Next, we used a digital twin heart model from a patient with postmyocardial infarction arrhythmias (Figures 1D–1F) based on preablation cardiac late gadolinium enhanced magnetic resonance imaging, and gray zone myocardium with remodeled ionic current properties and slowed conduction.^2^ Core scar was made inexcitable. VT was induced by programmed stimulation to identify the exit site of the arrhythmia. Next, we prolonged repolarization of the nonscarred myocardium adjacent to the exit site by 50 ms and repeated the stimulation protocol for VT induction. This only resulted in a single spontaneous premature ventricular beat, after which arrhythmic quiescence ensued.

Our findings show the potential of local prolongation of repolarization as a novel antiarrhythmic therapy in postmyocardial infarction patients. In the isolated heart and in the virtual patient heart, local prolongation of repolarization in myocardium with fractionated potentials or containing the VT exit site was effective in preventing VT. If multiple exit sites had been present, an unsuccessful intervention would have been inconclusive because the arrhythmias could have been sustained by a different exit site. When multiple exit sites are present, multiple regions need to be selectively targeted.

Site-specific therapy may prevent off-target effects of systemic therapy or unnecessary ablation of viable myocardium or the induction of new scars. Local delivery of repolarization prolonging drugs could be achieved by materials that support drug release, such as bioresorbable adhesive devices.^3^ Alternatively, long-term local prolongation of repolarization could be achieved using cardiac gene therapy.^4^ The latter has evolved into a high-potential therapeutic strategy targeting various disorders. For long-term gene delivery, adeno-associated virus vectors have emerged as the most potent system and are now being tested in various clinical trials primarily targeting heart failure.^5^

Our study is limited by the results of a single experiment and a single *in silico* observation. Thus, it can be argued that the outcome of the experiment was coincidental. However, we have shown that a pig heart is very stable during Langendorff perfusion and that the heart can serve as its own control for interventions such as infusion of sotalol. Second, we showed that the effect of sotalol was mediated by repolarization prolongation. Also, the results are confirmed by the patient-specific simulations, in which confounding variability is absent, providing translational value. Thus, a single experiment can be valuable to establish proof of concept. Future studies should test our intervention in the setting of more complex reentrant pathways with multiple exit sites.

In conclusion, site-specific targeted antiarrhythmic therapy carries promise for nondestructive, mechanism-based treatment of arrhythmic substrates, to a degree not attained with drugs or ablation and without their side effects.
